# Redox imbalance in Crohn's disease patients is modulated by Azathioprine

**DOI:** 10.1080/13510002.2021.1915665

**Published:** 2021-04-21

**Authors:** Mohammad Javad Tavassolifar, Mostafa Changaei, Zahra Salehi, Fatemeh Ghasemi, Moslem Javidan, Mohammad Hossein Nicknam, Mohammad Reza Pourmand

**Affiliations:** a Immunology Department, School of Medicine, Tehran University of Medical Sciences, Tehran, Iran; b Department of Pathobiology, School of Public Health, Tehran University of Medical Sciences, Tehran, Iran

**Keywords:** Redox, Crohn's disease, inflammatory bowel disease, reactive oxygen species, peripheral blood mononuclear cell

## Abstract

**Background:**

Crohn's disease (CD) is a chronic inflammatory disease without a specific cause. Inflammation in these patients can disturb the oxidants/antioxidants balance and results in oxidative stress that plays a destructive role. This study aimed to evaluate the gene expression of *sod1*, *sod2*, *cat*, *nrf2* and *gp91phox* in CD patients before and after Azathioprine (Aza) consumption.

**Method:**

Peripheral bloodmononuclear cells (PBMCs) were separated from CD patients (*n*= 15, mean age = 33.6 ± 1.8) before and after treatment with Aza and healthy controls (*n*= 15, mean age = 31.5 ± 1.2). The expression levels of *sod1*, *sod2*, *cat*, *nrf2* and *gp91phox* were measured in byusing real-time qRT-PCR technique.

**Result:**

The expression levels of *gp91phox* (*P-*value < 0.001), *cat* (*P-*value < 0.05), *sod1* (*P*-value < 0.001), *nrf2* (*P*-value < 0.001) were significantly increased compared to control group. Following treatment with Aza, the decreased expression levels of *gp91phox* (*P-*value < 0.05), *cat* (*P-*value < 0.05), *sod1*(*P*-value < 0.001) and *nrf2* (*P*-value < 0.001) were observed in CD patients.

**Conclusion:**

Overall, our results showed that prescription of Azathioprine can lead to the altered expression of redox system-related genes in patients with CD.

## Introduction

Crohn’s disease (CD) is a chronic inflammatory bowel disease (IBD) that affects different parts of the gastrointestinal tract, frequently terminal ileum [1]. CD’s main clinical manifestations include fever, fatigue, abdominal pain, diarrhea, fistula, and anal lesions [2]. With a rapidly increasing trend worldwide, CD incidence has been estimated at 0.06–29.3 per 100,000 [3]. Decades of basic and clinical studies increased our knowledge about molecular mechanisms underlying CD, but its etiology is still unclear [4].

Various genetic and environmental factors have been speculated to play a significant role in CD pathogenesis [5]. Inflammatory conditions that are mainly immunologically mediated thought to underlie the development of CD [6]. Altered host immune responses against intestinal microbiota followed by impaired epithelial-mucosal barrier result in releasing many inflammatory agents and subsequent tissue injury in CD [7–9]. Of note, inflammation increases inflammatory cytokines production such as IL-1β, IL-6, TNF-α and IFN-γ and promotes oxidative stress [10,11]. As a result, significantly augmented the GP91PHOX activity during the immune responses could be noticed [12].

GP91PHOX, also called NOX-2 is a transmembrane protein and acts as a catalytic subunit of NADPH oxidase. Activation of GP91PHOX leads to the generation of superoxide onions (O2^•-^) through electron transfer from NADPH to O2. This process is a crucial step in producing reactive oxygen species (ROS) [13]. Redox-active molecules such as ROS have crucial functions in eradicating pathogens and regulating inflammatory processes [14]. Exceed production of ROS causes cellular damage and aggravates autoimmune diseases’ pathobiology [15,16]. In normal situations, antioxidant proteins neutralize free radicals and inhibit ROS production by preventing cellular damages and tissue injury triggered by oxidative stress. For instance, superoxide dismutases (SODs) could convert O2^•-^ to H2O2. Then, catalase reduces H2O2 to water [17]. As a transcription factor, Nuclear factor erythroid 2-related factor 2 (NRF2) binds to antioxidant response element (ARE) sequences in the promoter of antioxidant enzymes genes, including NADPH quinone oxidoreductase1 (NQO-1), catalase (CAT), and SODs [18]. Dysregulation of NRF2 has been documented in many autoimmune disorders [19–21].

Since the elevated levels of ROS along with impairment of the antioxidant defense system result in cellular damages and tissue injury. In the present study, we aimed to evaluate the expression of redox system genes including *sod1*, *sod2*, *cat*, *gp91phox* and *nrf2* in patients suffering from CD. Besides, we examined the effect of Azathioprine as an immunosuppressant drug on oxidative stress levels in these patients.

## Material and method

### Study subjects

A longitudinal follow-up study was conducted on fifteen CD patients (female = 12, male = 3, mean age = 33.6 ± 1.8), referred to the Imam Khomeini General Hospital, Tehran University of Medical Sciences, Tehran, Iran. Diagnosis of CD was confirmed by clinical symptoms, laboratory evaluations, colonoscopy examinations, and histological criteria. Also, the disease severity is estimated according to the CD Activity Index (CDAI). Fifteen healthy persons (female = 11, male = 4, mean age = 31.5 ± 1.2) without a history of autoimmune and immunodeficiency disorders were also enrolled in the current study (Table 1). Neither CD patients nor healthy individuals used antibiotics, probiotics, and immune suppressive drugs at least in the three months before the study. According to the gastroenterologist's advice, Azathioprine was prescribed to CD patients (50 mg per day) after three months from the first sampling. Then, the second sample was collected.

**Table 1.** Characteristics of CD patients and healthy controls.

	Sex (Female/Male)	BMI	Age/years	Severity Score	WBC	CRP	ESR	Hem	Hct
CD patients (*n *= 15)	12/3	22.3 ± 1	33.6 ± 1.8	186 ± 14.7	7560 ± 416.7	18 ± 3.3	24 ± 5.5	12.5 ± 0.3	42 ± 1
Controls (*n *= 15)	11/4	31.5 ± 0.5	31.5 ± 1.2	–	–	–	–	–	–

BMI, body mass index; WBC, white blood cell; CRP, C-reactive protein; ESR, erythrocyte sedimentation rate; Hem, hemoglobin; Hct, hematocrit.

All patients and controls were of Iranian origin. Voluntary participants were informed about the study's procedure, and informed consent was obtained from them. The study was approved by the ‘Ethics Committee of Tehran University of Medical Sciences IR.TUMS.SPH.REC.1398.060' and conducted in accordance with the Declaration of Helsinki.

### RNA extraction

A whole blood sample was collected from all CD patients and controls. For isolation of peripheral blood mononuclear cells (PBMCs), whole blood was diluted 1:1 with PBS, the mixture gently layered over the Ficoll density-gradient media (Lymphodex Inno, Germany), and then centrifuged at 1000 g for 20 min. The layer containing PBMCs was transferred to a new tube, washed three times, and prepared for the RNA extraction procedure. According to the manufacturer's instructions, total RNA was extracted with an RNX-Plus extraction kit (RN7713C, sinaclon, Iran). RNA concentration and purity were checked by 260/280 nm absorbance ratio using NanoDrop (Thermo Fisher).

### Real-time PCR

First, total RNA was treated with DNase I, RNase free (Thermo Fisher Scientific, U.S.A.) to avoid genomic DNA contamination. Then, using a first-strand cDNA synthesis kit (Thermo Fisher Scientific, U.S.A.), total RNA was reverse transcribed into complementary DNA (cDNA) in a reaction primed by a random hexamer according to the manufacturer's instructions. The mRNA levels of *cat*, *gp91phox*, *nrf2*, *sod1* and *sod2* were measured by RT-qPCR with appropriate primers (Table 2). Amplification was performed on Applied Biosystem and revealed with SYBR-Green master mix (Ampliqone, Denmark). The cycling condition was performed for desired genes, as mentioned in Table 2. Melting curve analysis indicated no primer-dimers and non-specific products in the assay [22]. Data were analyzed, and threshold cycle (Ct) values were determined. The average expression levels of mRNA were normalized to 18 s rRNA using the 2^−ΔΔCt^ method.

**Table 2.** Primers sequences and cycling condition used for gene expression analysis through real-time qRT-PCR.

Gene	Amplicon Length (bp)	Forward primer (5'→3')	Reverse primer (3'→5')	Cycling condition
*gp91-phox*	106	CTGGAAACCCTCCTATGACTTG	GTGATGACCACCTTCTGTTGAG	40 Cycles, 59°C
*catalase*	238	TGCTGAATGAGGAACAGAGGAA	CCTCACAGATTTGCCTTCTCC	40 Cycles, 60°C
*nrf2*	204	CCATTCCTGAGTTACAGTGTCT	CTGTGGAGAGGATGCTGC	40 Cycles, 58°C
*sod1*	181	AGCGAGTTATGGCGACGAAG	CAGCCTGCTGTATTATCTCCA	40 Cycles, 59°C
*sod2*	144	CTCAGGTTGGGGTTGGCT	TGAAGGTAGTAAGCGTGCTCC	40 Cycles, 57°C
*18s rRNA*	151	GTAACCCGTTGAACCCCATT	CCATCCAATCGGTAGTAGCG	40 Cycles, 59°C

bp, base-pair; NRF2, nuclear factor erythroid 2-related factor 2; SOD1, superoxide dismutase-1; SOD2, superoxide dismutase-2.

### Statistical analysis

Data were analyzed using SPSS statistical software (version 19.0, IBM, New York, U.S.A.). One-way ANOVA followed by turkey’s post hoc test was applied to compare data from patients and control groups. All tests were two-tailed. *P*-value < 0.05 was considered statistically significant.

## Results

**Figure 1.**
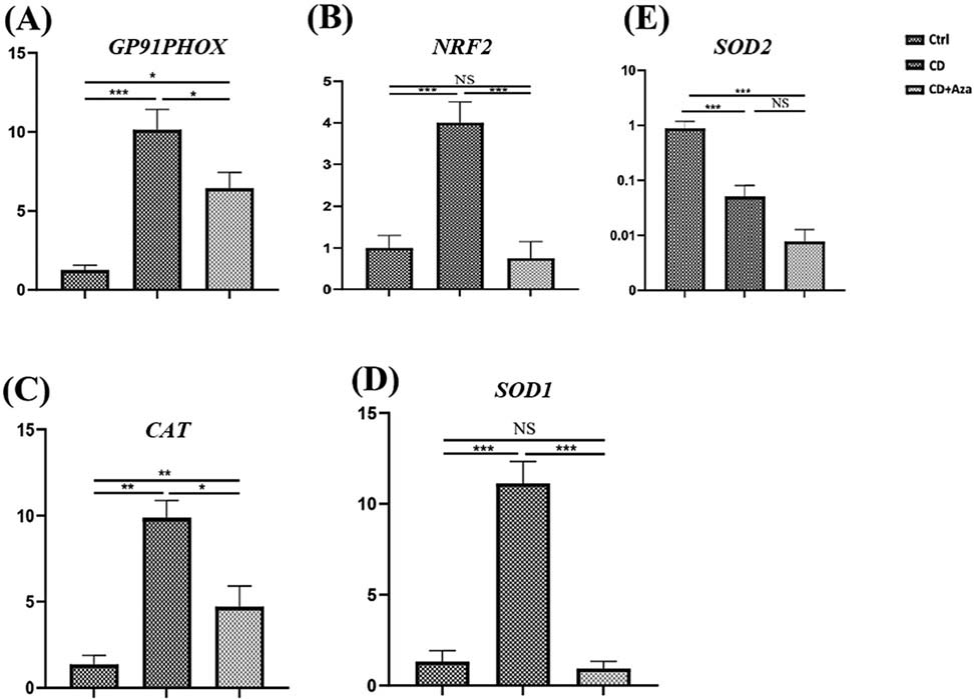
Elevated expression of oxidative stress markers in patients with CD (CD). Fold changing expression of GP91PHOX (A), NRF2 (B), CATALASE (C), SOD1 (D), SOD2 (E) were measured in the PBMCs of CD patients before and after prescription of Azathioprine (Aza). Data are shown as average ± standard deviation (SD). Comparison between CD, Azathioprine receiving CD patients (CD + Aza) groups and healthy controls (Ctrl) was performed by One-way ANOVA and Turkey’s post hoc test. *P-value < 0.05, **P-value < 0.01, ***P-value < 0.05, NS: non-significant.

To determine whether *the gp91phox* expression level was altered in CD, we investigated mRNA levels of *gp91phox* using a quantitative RT–PCR assay. Gene expression analysis showed a significant increase in *gp91phox* mRNA levels in CD patients than in the control group (8.35 ± 1.88 vs. 1.25 ± 0.29; *P*-value < 0.001). Furthermore, mRNA levels of *gp91phox* were measured after the prescription of Azathioprine to patients, and the results demonstrated that expression of *gp91phox* significantly decreased to normal levels (6.44 ± 0.80 vs. 1.25 ± 0.29; *P*-value < 0.05) (Figure 1(A)).

Redox regulation is enzymatically controlled. CAT, SOD1 and SOD2 have essential roles in scavenging free radicals [17]. The imbalance between radical-generating and radical-scavenging systems considered as a potential mechanism in the pathophysiology of autoimmune disorders [23]. Therefore, we measured mRNA levels of *cat*, *sod1*, *sod2* and up-stream transcription factor *nrf2* in CD patients. The results indicated that increased expression levels of *cat* (9.86 ± 2.5 vs. 1.36 ± 0.52; *P*-value < 0.05), *SOD1* (12.76 ± 2.75 vs. 1.32 ± 0.33; *P*-value < 0.001), and *nrf2* (18.27 ± 4.85 vs. 2.4 ± 0.74; *P*-value < 0.001) in patients compared with controls (Figure 1(B–D)). Remarkably, treatment with Azathioprine resulted in a significant decrease in mRNA levels of *cat* (9.86 ± 2.5 vs. 4.7 ± 1.1; *P*-value < 0.05), *sod1* (12.76 ± 2.75 vs. 0.92 ± 0.52; *P*-value < 0.001) and *nrf2* (18.27 ± 4.85 vs. 2.04 ± 0.71; *P-*value < 0.001) in CD patients. Interestingly, expression levels of *sod2* were significantly lower in patients compared with controls (0.05 ± 0.01 vs. 0.87 ± 0.22; *P*-value < 0.001) which showed decreased expression in patients compared to controls after treatment with Azathioprine. However, the difference was not statistically significant (0.05 ± 0.01 vs. 0.007 ± 0.002; *P*-value > 0.05) (Figure 1(E)).

## Discussion

Previous studies have shown that O2^•-^ produced by GP91PHOX can lead to cell damage and tissue injury in many autoimmune diseases [24]. There is cumulative evidence regarding the roles of oxidative stress in CD's pathogenesis [25–27]. Superoxide radical is the progenitor of ROS, including OH^-^, RO^-^, ROO^-^ and H2O2. Furthermore, superoxide radicals can react with highly reactive nitric oxide (NO^-^) molecules to generate the peroxynitrite anion (ONOO^-^), or so-called reactive oxygen and nitrogen species (RONS) [28]. These highly reactive molecules can contribute to CD pathogenesis through DNA damage and lipid oxidation [28]. In the present study, we observed higher mRNA levels of oxidative stress-related markers in CD patients than in the healthy group, which reduced following Azathioprine treatment.

Here, we showed a significantly higher expression level of *gp91phox* in CD patients than in healthy individuals. Bao et al. showed that GP91PHOX contributes to inflammation progression in a mouse model of acute colitis. Their results indicated that *gp91phox*^-/-^ mice produce lower levels of pro-inflammatory cytokines compared with WT mice [29]. Also, increased expression of *gp91phox* has been observed in colitis's rat model after stimulation with serotonin [30].

In physiological condition, endogenous anti-oxidative defense components such as SODs and CAT eliminate excess ROS to prevent oxidative stress-related damage [31]. There are three different superoxide dismutase genes in the eukaryotic genome: *sod1* and *sod3* encode cytoplasmic and extracellular Cu/Zn SOD, respectively and *sod2* encodes mitochondrial Mn-SOD [32,33]. Our data revealed significantly higher mRNA levels of *sod1* and *cat* in CD patients compared to healthy controls. But, lower level of *sod2* was observed in patients. Similarly, Dincer et al. suggested augmented expression of *sods* in blood cells of IBD patients [34]. In contrast, others reported decreased SODs and CAT activity in CD and ulcerative colitis [35,36]. It seems the lower *sod2* levels in Crohn's patients are related to impaired mitochondrial structure and function. Nazli et al. and Söderholm et al. have reported that isolated enterocytes from patients with IBD exhibit swollen mitochondria with abnormal cristae [37,38]. Also, Rodenburg et al. demonstrated an abnormal mitochondrial structure in experimental mice models of colitis [39]. Cellular stress and bioenergetic failure are indicative of these morphological shifts. Indeed, patients with CD have decreased ATP levels and short-chain fatty acids (SCFA) β-oxidation deficiencies. However, it is uncertain if the reported changes in the mitochondrial structure and function are due to disease or play a role in pathogenesis [40].

NRF2 is recognized as a critical regulator that controls the transcriptional activation of genes involved in endogenous antioxidant biosynthesis [41]. It binds to ARE sequences in the promoter of target genes [18]. In normal oxidative conditions, NRF2 is constitutively ubiquitinated by Kelch-like ECH-associated protein 1 (Keap1) and Cullin-3 E3 ligase and degraded by the 26S proteasomal pathway [42]. Under oxidative stress and exposure to ROS, NRF2 translocates to the nucleus and induces transcriptional activation of endogenous antioxidant defense system genes such as *Sods* and *cat* [18,43,44]. Here, we indicated higher levels of *nrf2* in CD patients compared to controls. These findings suggested that CD patients show higher levels of NRF2 dependent antioxidants (SOD1 and CAT) in response to increased ROS molecules. However, increased mRNA levels of *sod1* and *cat* cannot explain the activity function of their proteins.

Azathioprine is a purine analog that commonly use to manage IBD, such as CD [45]. The main mechanism of Azathioprine that contributes to immunosuppression is its association with suppression of DNA replication. Hypoxanthine-guanine phosphoribosyl transferase (HPRT) and thiopurine methyltransferase (TPMT) enzymes convert Aza to active metabolites, including mercaptopurine (6-MP) and thioguanine (6-TGN) which inhibit the synthesis of an essential purine for DNA and RNA production [46]. Consequently, Aza may result in decreased expression of *gp91phox, sod2, nrf2* and its downstream genes including *cat* and *sod1* through suppressing purine metabolism in white blood cells.

On the other hand, CD is characterized by augmented inflammatory responses. Therefore, anti-inflammatory treatments such as Azathioprine are widely used to reduce CD symptoms [45]. We found that prescribing anti-inflammatory drugs such as Azathioprine could decrease *gp91phox* expression in the patients’ group to a normal level. Also, the expression of *sod2*, *nrf2* and its downstream genes including *sod1* and *cat* decreased following Azathioprine treatment. It has been demonstrated that oxidative stress results in abnormal immune responses and over-expression of pro-inflammatory cytokines including TNF-α, interleukin (IL)-1 and IL-6 which have essential roles in the progress and maintenance of CD pathogenesis [47]. Further investigations have also shown inflammatory cytokines promote over-expression of *gp91phox* through the NF-κB pathway as well as inflammasome signaling [48]. According to a study by Abais et al., NRF2 has a protective effect against inflammatory cytokines in a mouse model of chronic colitis [49]. In line with our research, long-term prescription of anti-inflammatory drugs such as Azathioprine has been shown to have an adverse effect on NRF*2* expression and it is associated with increased oxidative stress [50].

## Conclusion

The results of this study showed that patients with CD tend to show increased expression of *gp91phox, sod1* and *cat* in the NRF2 dependent pathway. Also, we showed immunosuppressant drugs such as Azathioprine may reduce oxidative stress-related genes in patients with CD, most likely through interrupting DNA and RNA biosynthesis and decreasing pro-inflammatory cytokines such as TNF-α, IL-1 and IL-6. As a limitation, we were unable to assess the enzyme activities and protein levels. So, as a suggestion, further studies in this field may contribute to a better understanding of underlying mechanisms of CD pathogenesis.
